# Periodic thermomechanical modulation of toll-like receptor expression and distribution in mesenchymal stromal cells

**DOI:** 10.1557/s43579-021-00049-5

**Published:** 2021-07-08

**Authors:** Xun Xu, Yan Nie, Weiwei Wang, Nan Ma, Andreas Lendlein

**Affiliations:** 1 Institute of Active Polymers and Berlin-Brandenburg Center for Regenerative Therapies, Helmholtz-Zentrum Hereon, 14513 Teltow, Germany; 2 Institute of Chemistry, University of Potsdam, 14476 Potsdam, Germany; 3 Institute of Chemistry and Biochemistry, Freie Universität Berlin, 14195 Berlin, Germany

**Keywords:** Actuation, Antiviral, Biomaterial, COVID-19, Shape memory

## Abstract

**Abstract:**

Toll-like receptor (TLR) can trigger an immune response against virus including SARS-CoV-2. TLR expression/distribution is varying in mesenchymal stromal cells (MSCs) depending on their culture environments. Here, to explore the effect of periodic thermomechanical cues on TLRs, thermally controlled shape-memory polymer sheets with programmable actuation capacity were created. The proportion of MSCs expressing SARS-CoV-2-associated TLRs was increased upon stimulation. The TLR4/7 colocalization was promoted and retained in the endoplasmic reticula. The TLR redistribution was driven by myosin-mediated F-actin assembly. These results highlight the potential of boosting the immunity for combating COVID-19 via thermomechanical preconditioning of MSCs.

**Graphic abstract:**

Periodic thermal and synchronous mechanical stimuli provided by polymer sheet actuators selectively promoted the expression of SARS-CoV-2-associated TLRs 4 and 7 in adipose-derived MSCs and recruited TLR4 to Endoplasmic reticulum region where TLR7 was located via controlling myosin-mediated F-actin cytoskeleton assembly.
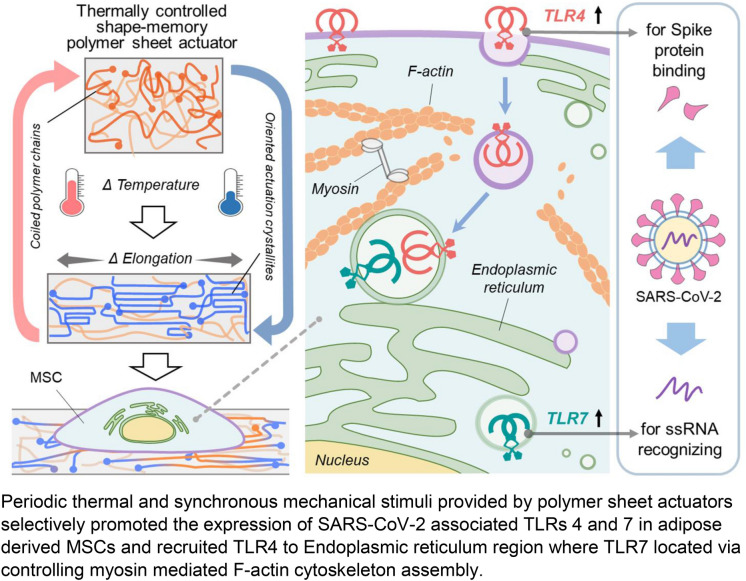

## Introduction

Coronavirus disease 2019 (COVID-19), which refers to a severe acute respiratory syndrome caused by the infection of SARS-CoV-2 virus,^[1]^ has affected over 100 million individuals and results in 2.5 million deaths globally during the fast-evolving pandemic. The symptoms of COVID-19 range from being asymptomatic and mild to severe. Although the mortality rates from different regions and countries are diverse, the severe cases with a high rate of death are observed mainly in elderly patients and those with underlying diseases, who can hardly retain a proper immune reaction against virus due to the immunosenescence.^[2]^ As the distinctive feature of severe COVID-19, the poor protective type-1 interferon (IFN)-mediated antiviral immunity with excessive pro-inflammatory cytokines indicates the participation of host immune system in COVID-19 pathogenesis.^[3]^ Nowadays, intravenous infusion of human mesenchymal stromal cells (MSCs) is emerging as a substantial therapeutic approach for COVID-19 pandemic, which mainly relies on their homing, regenerative capacity, low immunogenicity, and immunomodulatory function for supporting small capillaries, protecting alveolar epithelial cells and counteracting fibrosis of the injured respiratory system.^[4]^

Toll-like receptors (TLRs) are typical sensors and responders, which are able to evoke innate immune responses against various foreign organisms including viruses by the production of pro-inflammatory cytokines and type-1 IFNs.^[5]^ TLRs are also crucial for regulation of migration, proliferation, differentiation, and immune polarization of MSCs.^[4]^ Human TLRs can be categorized into ten subfamilies regarding their initial subcellular locations, as TLRs 1, 2, 4, 5, 6, and 10 are existent on the cell membrane, while TLRs 3, 7, 8, and 9 are located intracellularly especially on endoplasmic reticula (ER).^[6]^ TLRs 3, 4, 7, 8, and 9 are typically associated with viral clearance by recognition of their nucleic acids or envelope proteins. TLR3 identifies viral double-stranded RNA (dsRNA) and TLR9 recognizes viral DNA containing unmethylated CpG islands.^[5]^ SARS-CoV-2, the pathogen of COVID-19, is a novel emerging beta-coronavirus containing single-stranded RNA (ssRNA) genome. Recent studies on COVID-19 have suggested that the initiation of the SARS-CoV-2 infection is governed not only by ACE2/TMPRSS2 receptor binding, but also by the recognition of TLR4 on cell surface with its Spike protein^[7]^ and TLR7 intracellularly with its ssRNA.^[8]^ In addition, Spike protein of SARS-CoV-2 can bind to bacterial lipopolysaccharide, which is also the ligand for TLR4 stimulation.^[9]^

TLR expression profiles and cellular distributions are varying in MSCs from diverse cell sources and can be tuned via changing the culture conditions. For instance, TLR4 is missing in the cells propagated from Wharton’s jelly tissue, while the similar level of TLR3 is expressed in both bone marrow and Wharton’s jelly-derived MSCs. Once the cells are cultured under inflammatory environment in the presence of a cocktail of inflammatory cytokines, the level of TLR3 is increased only in Wharton’s jelly-derived MSCs.^[10]^ Cellular behaviors and functions of MSCs can be altered by the biochemical and biophysical signals as well as thermal and mechanical cues.^[11]^ Priming TLR4 on MSC surfaces with chemical compounds results in pro-inflammatory and antigen-presenting cell-like phenotypes leading to release of pro-inflammatory cytokines and recruitment of immune cells to raise the immune reactions. However, priming of TLR3 makes the switching of MSCs toward an anti-inflammatory phenotype to suppress immune response.^[12]^ It has been demonstrated that the mechanosensor TRPM7 and thermosensor TRPM8 are essential for mediating TLR cellular distribution and inflammatory responses.^[13,14]^ Both TRPM channel proteins are also found to be expressed in MSCs suggesting a potential role of thermomechanical stimulation on modulating SARS-CoV-2-associated TLRs.

Given the fact that there is recruitment of infused MSCs at the interface of lung alveoli and their surrounding capillaries,^[4]^ where respiratory airway structures are exposed to periodic heat exchange and mechanical stresses with respiratory cycles,^[15]^ thermally controlled cPCLBA (copolymer network containing crystallizable poly(ε-caprolactone) and amorphous poly(*n*-butylacrylate) segments) sheets with programmable actuation capacity were applied in the present study to autonomously generate, synchronize, and transfer the periodic thermomechanical stimuli to human adipose-derived MSCs. The cPCLBA polymer sheets can be programmed by implementing a scaffold formed by crystallites into the material, which defines the geometry of the movement. Within this internal scaffold chain, segments are oriented along the deformation direction and form the actuation units. These enable the stretching by crystallization-induced elongation and contraction upon melting of the actuation crystallites driven by entropy gain.^[11,16]^ Here, the proportion of different viral pathogenesis-related TLRs on and inside MSCs, the subcellular distribution of SARS-CoV-2-associated TLRs including TLR4 and TLR7, and their interactions as well as the underlying biological mechanism in response to thermomechanical stimuli were investigated.

## Experimental details

### Cell source and maintenance

Human MSCs, isolated from the adipose tissue of a single female donor, were purchased from Merck Millipore (Catalog number SCC038, Merck Millipore, Darmstadt, Germany). Cells were maintained at a constant temperature of 37°C in an incubator supplied with 5 vol% CO_2_ in StemMACS™ MSC Expansion Medium (Miltenyi Biotec, Bergisch Gladbach, Germany). The medium was changed every 2 days and the cells were passaged when the confluence reached 80%. MSCs at passage 3 with viability higher than 90% as determined with a Countess™ Automated Cell Counter (Thermo Fisher Scientific, Schwerte, Germany) were applied for all the experiments.

### Preparation of shape-memory polymer actuator sheets and generation of periodic thermomechanical stimuli

Synchronized thermal and mechanical stimuli were generated by the thermally controlled cPCLBA sheets with programmable actuation capacity. cPCLBA containing crystallizable poly(ε-caprolactone) and amorphous poly(n-butylacrylate) segments was synthesized, programmed, and punched into circular sheets with a diameter of 10 mm fitting 24-well cell culture plates according to the methods described in our previous report.^[11]^ Each well of the 24-well plates was filled with 0.8 mL StemMACS™ MSC Expansion Medium (Miltenyi Biotec, Bergisch Gladbach, Germany) to immerse the cell-laden polymer sheet and supplied with 5 vol% CO_2_ for cell maintenance. The periodic thermal fluctuation between 37 and 10°C was realized by computer-controlled thermochambers (Instec, Colordao, USA). For each cooling/heating cycle, the program was set to 8 min cooling (37 to 10°C) and 22 min holding at 10°C, together with 8 min heating (10 to 37°C) and holding at 37°C for 22 min. Non-programmed cell-laden polymer sheets placed in thermochambers were used for the group with thermal stimulus. A stretching device (MCB1, CellScale, Ontario, Canada) was used to generate the periodic mechanical stimulus to the polymer sheets at a constant temperature of 37°C. To have the same shape change behavior as inside the thermochambers, the polymer sheet was stretched to 10% elongation in each cycle. The time was set to 60 min for each stretching cycle, consisting of 8 min of stretching, 22 min holding, 8 min of relaxing, and 22 min holding. Polymer sheets at constant temperature (37°C) in the absence of thermomechanical stimuli were set as untreated groups.

### Flow cytometry

At indicated time points under different culture conditions, 1 million living cells were freshly harvested, fixed, and permeabilized using Inside Stain Kit (Miltenyi Biotec, Bergisch Gladbach, Germany) following the manufacturer’s guidance. Cells were then incubated directly with anti-TLR3-FITC (mouse IgG, anti-human, monoclonal, 1:10, Miltenyi Biotec, Bergisch Gladbach, Germany) and anti-TLR9-APC antibodies (rat IgG, anti-human, monoclonal, 1:20, Thermo Fisher Scientific, Schwerte, Germany), or anti-TLR4-APC (mouse IgG, anti-human, monoclonal, 1:20, Thermo Fisher Scientific, Schwerte, Germany) together with anti-TLR7-FITC antibodies (rabbit IgG, anti-human/mouse, polyclonal, 1:50, Thermo Fisher Scientific, Schwerte, Germany), or anti-IFN-α1 (mouse IgG, anti-human, monoclonal, 1:50, Miltenyi Biotec, Bergisch Gladbach, Germany) in dark at 4°C for 20 min. For intracellular IFN-α1 detection, cells were treated with 3 μg/ml Brefeldin A (Thermo Fisher Scientific, Schwerte, Germany) for one hour prior to fixation. Stained cells were detected by MACSQuant flow cytometer (Miltenyi Biotec, Bergisch Gladbach, Germany). Data analysis was performed by using “Flowjo” software (FlowJo, LLC, Ashland, USA).

### Laser scanning confocal microscopy

MSCs were fixed with 4 wt% paraformaldehyde, permeabilized with 0.5 vol% Triton X-100 and blocked with 3 wt% BSA using Image-iT Fixation/Permeabilization Kit (Thermo Fisher Scientific, Schwerte, Germany) according to the user manual. Cells were then incubated with antibodies including anti-TLR4-APC (mouse IgG, 1:20) and anti-TLR7-FITC antibodies (rabbit IgG, 1:50, Thermo Fisher Scientific, Schwerte, Germany) overnight at 4°C. ER was labeled with Alexa Fluor 594-conjugated Concanavalin A. F-actin cytoskeleton was stained by ActinRed 555 ReadyProbes. And the cell nuclei were stained with Hoechst 33342-based NucBlue Live ReadyProbes (all dyes for cellular compartments were purchased from Thermo Fisher Scientific, Schwerte, Germany). Super resolution confocal fluorescence imaging was carried out using LSM 780 microscope equipped with Airyscan detector (Carl Zeiss, Jena, Germany). Airyscan calculation and maximum intensity projection were performed for all the Z-stack image series of cells using Zen 10 software (Carl Zeiss). The mean fluorescent intensity (MFI) of F-actin and the proportions of colocalized TLRs or TLRs with ER/F-actin were measured by ImageJ software with Bio-Formats Importer plugin (National Institutes of Health, USA).

### Inhibition of myosin II activity

Inhibition of myosin II-mediated F-actin assembly was carried out by applying the selective myosin light chain kinase (MLCK) inhibitor ML7 hydrochloride (5 µM working concentration; Bio-Techne GmbH, Wiesbaden, Germany) to MSCs for 16 h prior to immunostaining.

### Statistical analysis

Statistical analysis was performed using GraphPad Prism (Version 8, GraphPad Software, Inc., USA). Multiple comparison of means in more than three groups was performed by one-way ANOVA with post hoc Tukey test. The significance of the difference between untreated and stimulated groups or between MLCK inhibitor (-) and MLCK inhibitor (+) with the same stimuli was determined using an independent two-tailed, unpaired Student’s *t*-test. A *p* value < 0.05 was considered to be statistically significant. The number of repetitions for each experiment was indicated in the figure legends, with all data presented as mean ± standard deviation.

## Results and discussion

### Periodic thermomechanical stimuli specifically promote the expression of SARS-CoV-2-associated TLRs

**Figure 1:**
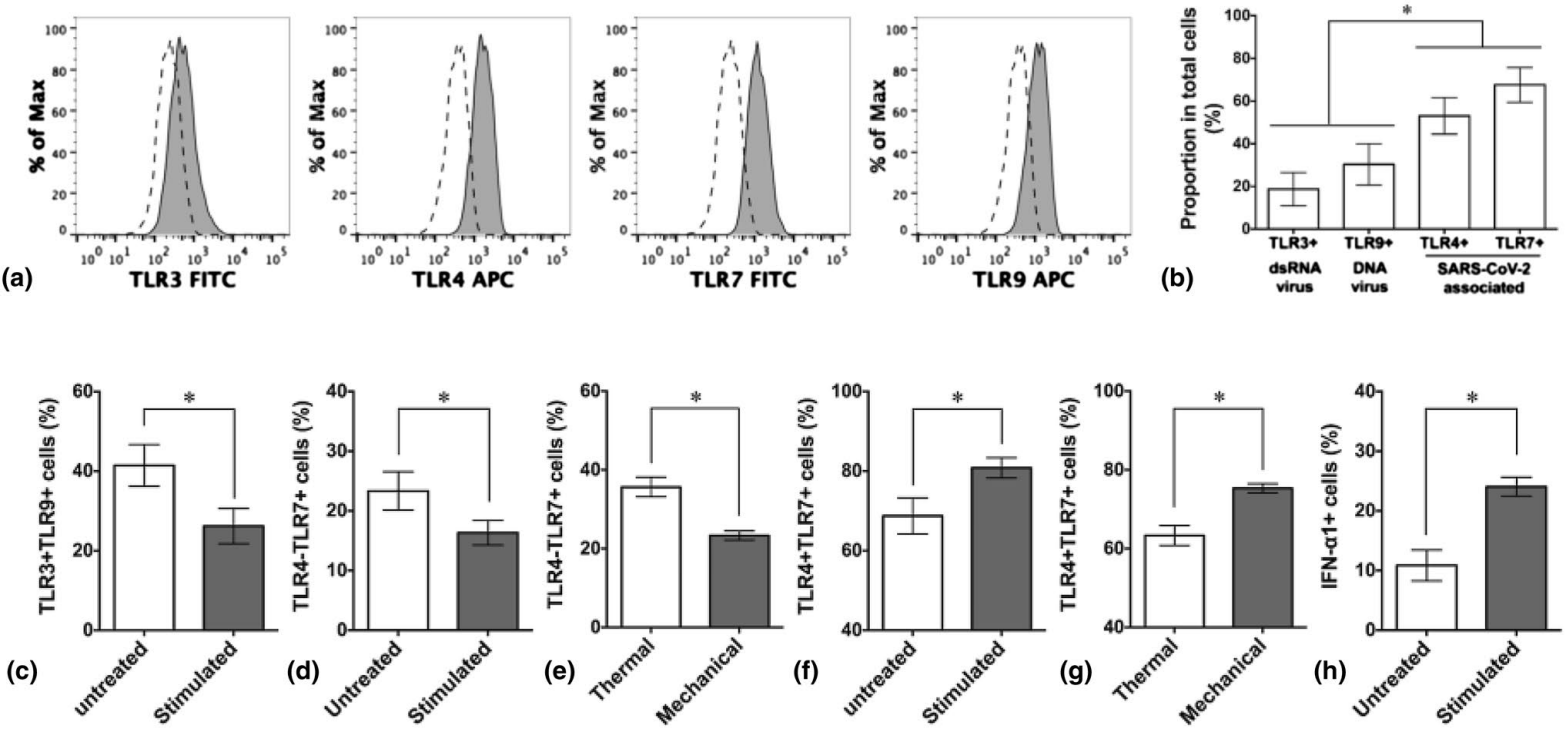
Expression of virus-associated TLRs in human adipose-derived MSCs with and without periodic thermomechanical stimulation. Representative flow cytometry histograms (a) and the expression profile (b) of viral infection-associated TLRs 3, 4, 7, and 9 in MSCs on day 0 before cell seeding on cPCLBA sheets (* *p* < 0.05, n = 3 independent samples, one-way ANOVA with Tukey test). (c) Quantitative analysis showing the alteration of the level of non-ssRNA viral infection-associated TLRs (TLR3 + TLR9 +) under thermomechanical stimulation. Subpopulation of SARS-CoV-2-associated TLRs (TLR4-TLR7 + and TLR4 + TLR7 +) in response to periodic thermomechanical stimulation (d, f) or individual stimulus (e, g). Quantification of IFN-α1 + MSCs with and without thermomechanical stimulation (h). For (c–h), * *p* < 0.05, *n* = 3 independent samples, unpaired Student’s *t*-test.

To access the profile of viral infection-associated TLRs in human adipose-derived MSCs and to understand the probability of boosting the therapeutic efficiency of COVID-19 via specific activation of proper TLRs, we harvested and analyzed the proportion of cells expressing virus-associated TLRs 3, 4, 7, and 9 under normal culture condition from tissue culture plates immediately before seeding them on the cPCLBA polymer sheet. TLRs 7 and 8 share structural homology and sense the same type of viral RNA.^[5]^ Here, TLR7 was selected as a representative for ssRNA virus-associated TLR [Fig. 1(a)]. Without priming by any viral pathogens, a significant high amount of MSCs that expressed SARS-CoV-2-associated TLRs including TLR4 (53 ± 9%) and TLR7 (68 ± 8%) was detected, while cells expressed TLR3 (19 ± 8%) and TLR9 (30 ± 10%) were kept at relatively low levels [Fig. 1(b)]. Our results are in agreement with the finding that TLR4 is highly expressed but are partially inconsistent with the same report showing a completely missing TLR7 mRNA in adipose-derived MSCs.^[10]^ This may be explained by the influences of distinct culture condition in different labs, the donor source of original adipose tissues, senescent status, and decreased level of IL-6 secreted by MSC themselves.^[10]^

The cyclic stretching of culture substrates together with respiratory epithelial cells can enhance their expression of TLR4.^[17]^ Mechanical ventilation modulates TLR3 signaling^[18]^ and there are connections of mechanosensor TRPM7 to TLRs.^[13]^ Moreover, the TLR4 expression on immune cells is shown to be promoted by high body temperature.^[19]^ Here, to further explore the effect and specificity of periodic thermomechanical stimulation on strengthening SARS-CoV-2-associated TLRs, we cultured cells on cPCLBA sheets for 4 days at constant temperature and under thermomechanical stimulation and divided the cell population into a dsRNA/DNA virus-associated group (TLR3 + TLR9 + cells) and a ssRNA SARS-CoV-2-associated group (TLR4 + TLR7 + cells) for flow cytometric analysis. The TLR3 + TLR9 + cells that are sensing dsRNA and DNA viral components were decreased from 41 ± 5% to 26 ± 4% [Fig. 1(c)]. Interestingly, TLR4 + TLR7- cells were non-detectable; TLR4-TLR7 + cells were reduced from 23 ± 3% to 16 ± 2%, while the TLR4 + TLR7 + cells were selectively expanded from 69 ± 5% to 81 ± 3% [Fig. 1(d, f)]. When looking at the individual effect of thermal or mechanical stimulus on SARS-CoV-2-associated TLRs, a decrease of TLR4-TLR7 + (36 ± 2% to 23 ± 1%) and an increase of TLR4 + TLR7 + MSCs (63 ± 3% to 75 ± 1%) under the mechanical stimulation were observed compared to those under the thermal stimulation [Fig. 1(e, g)]. The proportion of cells producing TLR-associated IFN-α1 was increased from 11 ± 3% to 24 ± 2% in response to the periodic thermomechanical stimulation [Fig. 1(h)].

### Colocalization of TLR4 and TLR7 in ER region in response to periodic thermomechanical stimulation

Subcellular distribution of surface TLRs can influence TLR-mediated immune response; surface-located TLRs only lead to an inflammatory reaction, but intracellular TLRs can trigger the releasing of both inflammatory cytokines and type-1 IFN.^[5]^ TLR4, which can provide the binding site for Spike protein of SARS-CoV-2, is typically expressed on the plasma membrane.^[7]^ Surface TLR4 can activate NF-kB signaling, triggering inflammatory response and even mediating cytokine storm with excessive pro-inflammatory cytokines.^[5]^ TLR7, an intracellular immune sensor capable of detecting viral ssRNA, is synthesized in the ER.^[20]^ Notably, TLR7 is able to induce the production of type-I IFNs that is missing post SARS-CoV-2 infection.^[3]^ As an organelle where viral entry, replication and assembly are taking place, ER that closely contacts with plasma membrane, endosome, lysosome, mitochondrion, Golgi apparatus, and nuclear membrane is highly associated with TLR intracellular trafficking.^[21,22]^ PRAT4A protein in the ER shows a critical role in maturation and plasma membrane presentation of TLR4.^[23]^ In addition, it is required for a proper folding and delivering of TLR7 to endosome containing IRF transcription factor to trigger type-1 IFN production.^[20]^ Another ER resident protein GP96 is required for stabilization and complex formation of TLR4 at ER region to produce type-1 IFNs.^[24]^ TRAF3 shuttled at ER and Golgi apparatus serves as a linker between TLRs and type-1 IFN induction.^[25]^ IRFs and NF-kB transcription factors play major role of antiviral response. TLR7 level are potentially elevated by activation of TLR4-mediated NF-kB signaling, which further enhances the type-1 IFN production.^[20]^

**Figure 2:**
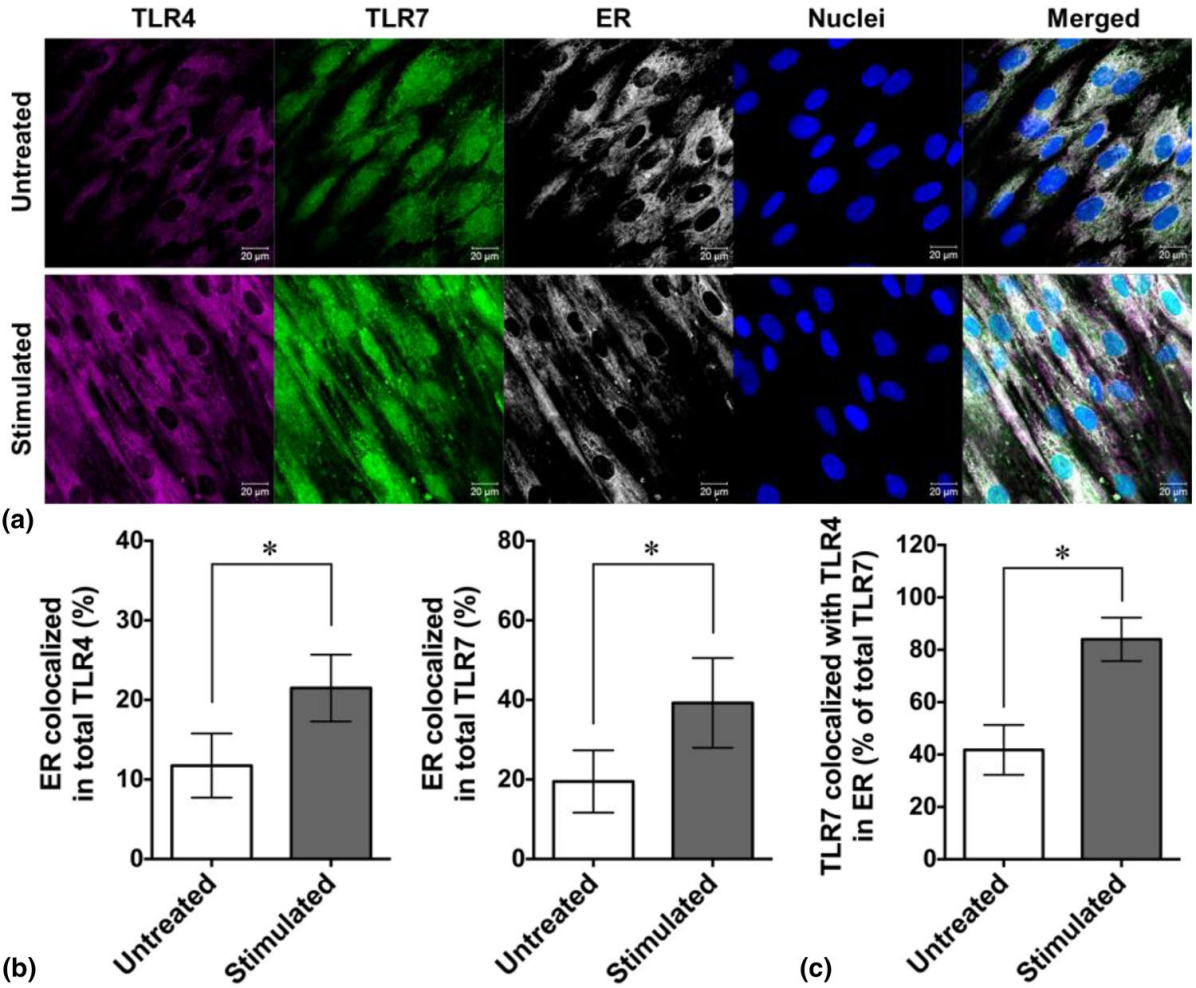
Subcellular distribution of SARS-CoV-2 associated TLRs in response to periodic thermomechanical stimulation. (a) Representative super resolution confocal fluorescence imaging showing subcellular distribution of TLR4, TLR7, endoplasmic reticula (ER), and cell nuclei after 3 days culture with and without thermomechanical stimulation. Scale bar: 20 μm. Confocal image-based quantitative analysis of the frequencies of TLR4 (left panel) and TLR7 (right panel) in ER regions (b), and the percentage of TLR4 colocalized TLR7 of total TLR7 in ER region (c) (* *p* < 0.05, *n *= 4 randomly selected images, unpaired Student’s *t*-test).

Here, after 3 days culture, enhanced colocalization of both TLR4 and TLR7 with ER could be observed [Fig. 2(a)]. A significantly larger amount of TLR4 (22 ± 2% vs. 12 ± 2%) was able to translocate into the ER regions upon periodic thermomechanical stimulation compared to the untreated cells grown at constant 37°C. Similarly, the proportion of TLR7 resided in ER was increased from 20 ± 4% to 39 ± 6% in the presence of thermomechanical stimuli [Fig. 2(b)]. TLR7 was shown to also accumulate in the nuclear region [Fig. 2(a)]. Although TLR nuclear distribution and their function in nucleus remains a mystery, we speculate that the nuclear TLR7 may have a possible role of interaction with nuclear transcription complex since ER membrane is connected to nuclear envelope and TLR7 contributes to transcription factor IRF-mediated type-1 IFN secretion. To clarify its nuclear function, further studies need to be performed. Interestingly, when focusing on the ER region, we found approximately that 85% of TLR7 was colocalized with TLR4 [Fig. 2(c)] suggesting an enhanced TLR crosstalk and synergy of type-1 IFN production in response to the periodic thermomechanical stimulation without activation from viral infection.

### Myosin alters TLR4 and TLR7 distribution via F-actin assembly in response to periodic thermomechanical stimulation

Mammalian cells largely rely on their cytoskeleton especially the F-actin filaments to realize the locomotion and mechanical interaction with local environment. Major cellular behaviors and functions are also correlated to contractile force transmission by the F-actin, which alters the nuclear shape, chromatin organization, and the translocation of transcription factors.^[26,27]^ The polymerization and sliding of F-actin associated with force generation has been demonstrated to be influenced by temperature change.^[28]^

**Figure 3:**
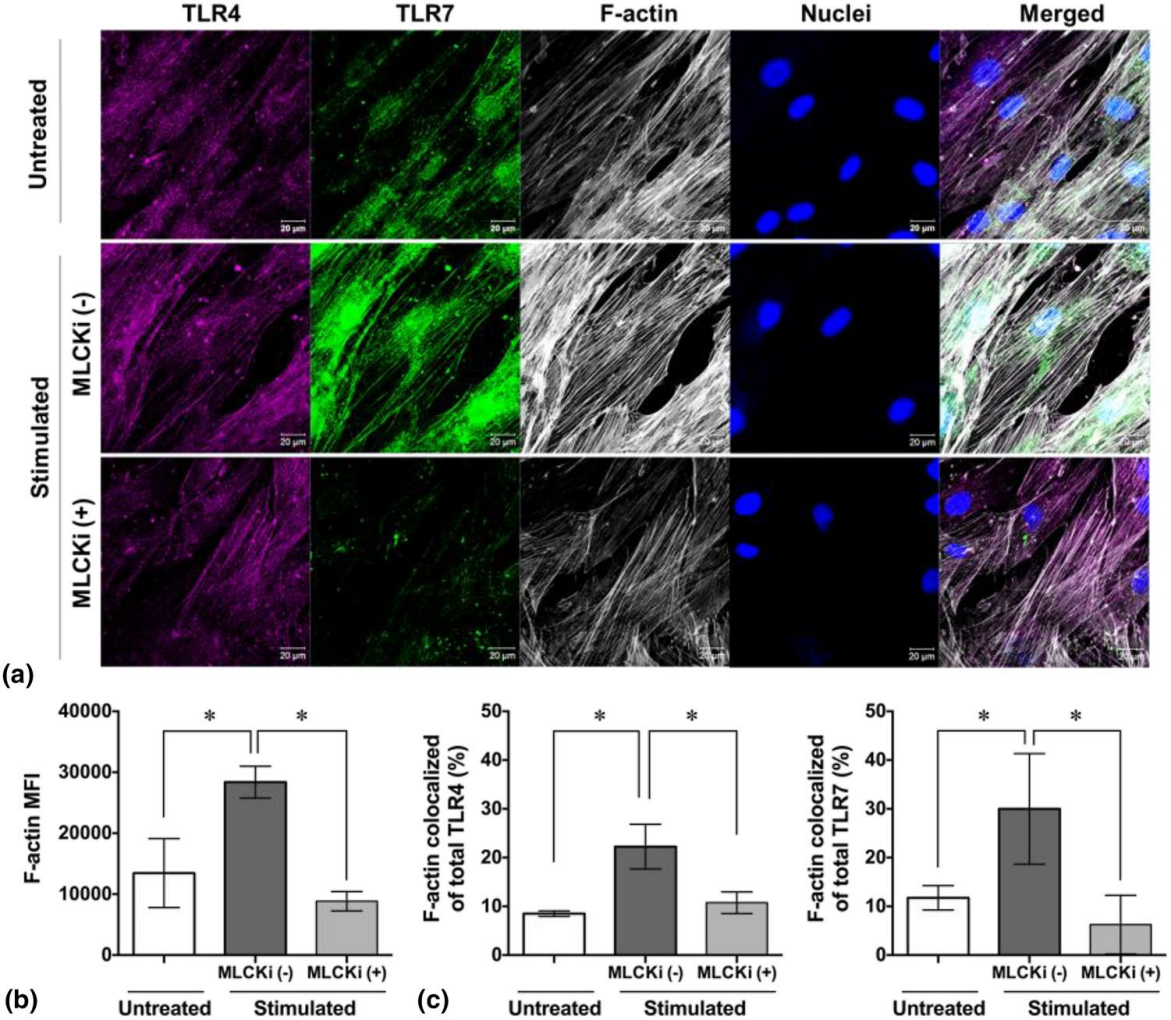
Alteration of F-actin cytoskeleton and subcellular distribution of SARS-CoV-2-associated TLRs in response to periodic thermomechanical stimulation. (a) Representative confocal imaging showing subcellular distribution of TLR4, TLR7, F-actin cytoskeleton, and cell nuclei after 4 days culture in response to thermomechanical stimulation with (+) and without (−) MLCK inhibitor (MLCKi). Scale bar: 20 μm. (b) Image-based quantification of mean fluorescence intensity (MFI) of F-actin in MSCs exposed to thermomechanical stimuli with and without MLCKi. (* *p* < 0.05, *n* = 4 randomly selected images, unpaired Student’s *t*-test). (c) Quantitative analysis of the frequencies of F-actin-colocalized TLR4 (left panel) and TLR7 (right panel) in their respective total populations in response to thermomechanical stimulation with and without MLCKi (* *p* < 0.05, *n* = 4 randomly selected images, unpaired Student’s *t*-test).

Importantly, actin dynamics and remodeling are shown to guide the internalization and endocytic vesicle trafficking in cellular compartments of variety of virus, cell receptors, and signaling proteins suggesting an underlying mechanism of regulating TLR subcellular distribution via F-actin cytoskeleton.^[29]^ Therefore, super resolution confocal images of SARS-CoV-2-associated TLRs and F-actin cytoskeleton were captured from different groups [Fig. 3(a)]. In situ position information from the images was then extracted for the following quantitative analysis. Here, after 4 days exposure to the periodic thermomechanical stimuli, the MFI, which reflects the F-actin amount and extent of actin polymerization, was doubled [Fig. 3(b)]. Both TLR4 and TLR7 colocalized along F-actin filaments were raised 2.6-fold upon stimulation [Fig. 3(c)].

Myosin, a molecular motor, is shown to not only mediate force generation, but also direct F-actin assembly and remodeling. The activity of its kinase MLCK can influence the actin-myosin interaction in majority of non-muscle cells including MSCs.^[30]^ Moreover, ER resident protein GP96, which facilitates ER translocation of TLRs and type-1 IFN production, is capable of controlling myosin dynamics.^[24]^ To explore the function of myosin in F-actin assembly and colocalization with TLRs in the presence of periodic thermomechanical stimuli, the selective inhibitor ML7 was applied to MSCs. As a result, the inhibition of MLCK markedly abolished the difference between the cells with and without thermomechanical stimulation (Fig. 3).

## Conclusions

The periodic thermomechanical stimulation realized by cPCLBA polymer sheets with programmable actuation capacity was able to specifically upregulate the expression of SARS-CoV-2-associated TLRs 4 and 7 in human adipose-derived MSCs and to recruit TLR4 at ER region where TLR7 resided in. By inhibition of the kinase activity of MLCK, we could explore the underlying mechanism that myosin altered the distribution of TLRs 4 and 7 via controlling F-actin assembly in the presence of thermomechanical stimuli. These findings highlight the possibility to strengthen the antiviral immunity against any variants of SARS-CoV-2 containing ssRNA by preconditioning of MSCs via periodic thermomechanical cues. This study also provides a valuable in vitro model for predicting the alteration of immune characteristics of infused MSCs in lung under the circumstances where periodic heat exchange and mechanical stresses continuously exist.

## Data Availability

Data will be made available on reasonable request.
